# Supplementary material for: Who participates in ‘participatory design’ of WASH infrastructure: A mixed-methods process evaluation
Source: PLOS Glob Public Health. 2025 Jun 13;5(6):e0003430. doi: 10.1371/journal.pgph.0003430 (PMC12165399; doi:10.1371/journal.pgph.0003430)
Supplement: S1 Table — (DOCX) [file pgph.0003430.s001.docx]

**S1 Table. Survey questions and response options for RISE participation, barriers to participation, experienced influence, and preferred influence**

| **1. Question: RISE activity participation** | “Which of the following RISE activities did you personally participate in?” |
| --- | --- |
| **Response options (Suva)** | Randomization workshop |
|  | Community co-design workshop for adults |
|  | Household consultation (during which you were asked about the placement of pipes, toilets, pump, wetlands, septic tanks, as well as maintenance costs) |
|  | Community co-design consent (during which connections were sprayed on the floor and wall and a picture was taken of the household representative with the marked connections) |
|  | Follow-up consent (during which someone from the household signed on a map of the settlement to confirm their agreement to participate in RISE) |
|  | Household data collection (during which RISE staff came to your house to take blood or stool or survey you or someone in your household) |
|  | Household data collection (during which RISE staff came to take measurements of your house, pipes, and floor levels) |
|  | Other |
|  | None |
|  | Don’t know |
|  | Refused to answer |
|  | Question was not asked |
| **2. Question: RISE activity participation** | “Which of the following RISE activities did you personally participate in?” |
| **Response options (Makassar)** | Randomization workshop |
|  | PANRITA workshop for adults |
|  | PANRITA workshop for youth |
|  | PANRITA workshop for children |
|  | Household consultation (during which you were asked about the placement of pipes, toilets, pump, wetlands, septic tanks, as well as maintenance costs) |
|  | Community co-design consent (during which connections were sprayed on the floor and wall and a picture was taken of the household representative with the marked connections) |
|  | Follow-up consent (during which someone from the household signed on a map of the settlement to confirm their agreement to participate in RISE) |
|  | Household data collection (during which RISE staff came to your house to take blood or stool or survey you or someone in your household) |
|  | Household data collection (during which RISE staff came to take measurements of your house, pipes, and floor levels) |
|  | Other |
|  | None |
|  | Don’t know |
|  | Refused to answer |
|  | Question was not asked |
| **3. Question: Barriers to participation** | “What was the main reason you did not participate in any RISE activities” |
| **Response options (both study countries)** | S/he was not aware of any of these activities |
|  | S/he was not invited by RISE to participate in any of these activities |
|  | S/he was unable to participate in any of these activities because s/he was too busy with work, housework, or school |
|  | S/he was unable to participate in any of these activities because her/his spouse or family would not allow it |
|  | S/he was unable to participate in any of these activities because s/he could not participate without assistance (e.g., visual, hearing, mobility difficulties) |
|  | S/he was unable to participate in any of these activities because s/he does not speak the language |
|  | S/he did not want to participate in any of these activities |
|  | We are renting our house |
|  | Other |
|  | Don’t know |
|  | Refused to answer |
|  | Question was not asked |
| **4. Question: Experienced influence** | “How much influence do you feel you and your household had over the RISE-related decisions that will affect your household, cluster, or settlement?” |
| **Response options (both study countries)** | No control/influence |
|  | A little control/influence |
|  | A lot of control/influence |
|  | Don’t know |
|  | Refused to answer |
|  | Question was not asked |
| **5. Question: Preferred influence** | “How much influence would you and your household have preferred to have over the RISE-related decisions that will affect your household, cluster, or settlement?” |
| **Response options (both study countries)** | No control/influence |
|  | A little control/influence |
|  | A lot of control/influence |
|  | Don’t know |
|  | Refused to answer |
|  | Question was not asked |
