# Supplementary material for: Who participates in ‘participatory design’ of WASH infrastructure: A mixed-methods process evaluation
Source: PLOS Glob Public Health. 2025 Jun 13;5(6):e0003430. doi: 10.1371/journal.pgph.0003430 (PMC12165399; doi:10.1371/journal.pgph.0003430)
Supplement: S2 Table — (DOCX) [file pgph.0003430.s002.docx]

**S2 Table. Qualitative in-depth interview and focus group discussion participant demographics**

| Individual Interview Participants | Indonesia residents (n=17) | | Fiji residents (n=12) | |
| --- | --- | --- | --- | --- |
|  | count or avg | % or range | count or avg | % or range |
| Number of settlements represented | 5 | – | 6 | – |
| Gender (Female) (n, %) | 13 | 76.5 | 8 | 66.7 |
| Age^1^ (avg, range) | 54.1 | 18.3-77.9 | 63.25 | 45-75 |
| Ethnicity (n, %) | 8 (Makassar)  6 (Bugis or Luwu)  3 (Other) | 47.1  35.3  17.6 | 5 (Indo-Fijian)  7 (iTaukei) | 41.7  58.3 |
| Religion (n, %) | 15 (Islam)  2 (Minority group) | 88.2  11.8 | 2 (Hindu)  5 (Christian)  5 (Unknown) | 16.6  41.7  41.7 |
| Person with a disability (n, %)  Yes  No  Unknown | 8  8  1 | 47.1  47.1  5.8 | 3  5  4 | 25.0  41.7  33.3 |
| Marital Status (n, %)  Married  Single/never married  Widower  Other  Unknown | 12  2  0  2  1 | 70.6  11.8  0  11.8  5.8 | 3  1  4  0  4 | 25.0  8.3  33.3  0  33.3 |
| Years lived in settlement (n, %)  Up to 5 years  5-10 years  More than 10 years  Whole life  Unknown | 1  4  2  2  8 | 5.8  23.5  11.8  11.8  47.1 | 4  1  6  0  1 | 33.3  8.3  50.0  0  8.3 |
| Individual Interview Participants | RISE staff (n=49) | | | |
|  | count or avg | | % | |
| Number of settlements represented | 6 | | – | |
| Gender (Female) (n, %) | 26 | | 53.1 | |
| Country office (n, %)  Indonesia  Fiji  Australia/USA | 13  13  23 | | 26.5  26.5  47 | |
| Role (n, %)  Project staff  Leadership | 40  9 | | 81.6  18.4 | |
| Focus Group Discussion Participants | Fiji (n=48) | | | |
|  | count or avg | | % or range | |
| # of settlements represented | 3 | | - | |
| Gender (Female) (n, %) | 23 | | 47.9 | |
| Age* (avg, range) | 42.5 | | 24-74 | |
| Ethnicity (n, %)  Fiji Indian  iTaukei  Rotuman  Unknown | 7  39  1  1 | | 14.6  81.2  2.1  2.1 | |
| ^1^ Missing some age data |  | |  | |
