# Supplementary material for: Who participates in ‘participatory design’ of WASH infrastructure: A mixed-methods process evaluation
Source: PLOS Glob Public Health. 2025 Jun 13;5(6):e0003430. doi: 10.1371/journal.pgph.0003430 (PMC12165399; doi:10.1371/journal.pgph.0003430)
Supplement: S3 Table — (DOCX) [file pgph.0003430.s003.docx]

| **S4 Table. Experienced and preferred influence among primary participatory design participants in Makassar** | | | | | | | | |
| --- | --- | --- | --- | --- | --- | --- | --- | --- |
|  | **Preferred Level of Influence** | | | | | |  |  |
|  | No influence | | A little influence | | A lot of influence | | **Total** | |
| **Experienced Level of Influence** |  |  |  |  |  |  |  |  |
| No influence | 37 | 58.7% | 14 | 22.2% | 12 | 19.1% | 63 | 100.0% |
| A little influence | 0 | 0.0% | 19 | 43.2% | 25 | 56.8% | 44 | 100.0% |
| A lot of influence | 3 | 2.2% | 30 | 21.7% | 105 | 76.1% | 138 | 100.0% |
| Missing observations:  7 (2.7%) missing from experienced level of influence  8 (3.1%) missing from preferred level of influence | | | | | | | | |
