# Supplementary material for: Who participates in ‘participatory design’ of WASH infrastructure: A mixed-methods process evaluation
Source: PLOS Glob Public Health. 2025 Jun 13;5(6):e0003430. doi: 10.1371/journal.pgph.0003430 (PMC12165399; doi:10.1371/journal.pgph.0003430)
Supplement: S5 Table — (DOCX) [file pgph.0003430.s005.docx]

| **S5 Table. Experienced and preferred influence among primary participatory design participants in Suva** | | | | | | | | |
| --- | --- | --- | --- | --- | --- | --- | --- | --- |
|  | **Preferred Level of Influence** | | | | | |  |  |
|  | No influence | | A little influence | | A lot of influence | | **Total** | |
| **Experienced Level of Influence** |  |  |  |  |  |  |  |  |
| No influence | 13 | 41.9% | 17 | 54.8% | 1 | 3.2% | 31 | 100.0% |
| A little influence | 1 | 1.9% | 44 | 81.5% | 9 | 16.7% | 54 | 100.0% |
| A lot of influence | 3 | 1.4% | 59 | 27.8% | 150 | 70.8% | 212 | 100.0% |
| Missing observations:  5 (3.1%) missing from both experienced level of influence and from preferred level of influence | | | | | | | | |
