# Supplementary material for: Who participates in ‘participatory design’ of WASH infrastructure: A mixed-methods process evaluation
Source: PLOS Glob Public Health. 2025 Jun 13;5(6):e0003430. doi: 10.1371/journal.pgph.0003430 (PMC12165399; doi:10.1371/journal.pgph.0003430)
Supplement: Attachment — Submitted filename: RISE participation.pdf [file pgph.0003430.s006.pdf]

The manuscript described a mixed-method approach to evaluate the participation by community members of RISE programs in two countries, with the intention to identify factors and barriers affecting participation. The report on this process evaluation is interesting and of importance, but several clarifications could be made to facilitate reader's understanding of the context.

1. To provide a background and context to the study, please give a description of the following concerns so that readers may get a general idea about the motivation to participate.
   i. What is the relative importance of this project among similar development programs in recent years?
   ii. What is the relationship between the agents of implementation and agents conducting the participatory processes?
   iii. What is the perception of local residents regarding the two questions above? What would motivate people to participate?
   iv. Please report on local residents' interest in the RISE program and perceived need for a water-sanitary service?
2. Related to the previous comment, the authors did not explain how the RISE program is going to have impact on the use of land, and how this was communicated with the residents before?
3. In assessing reach (p. 6): "We operationalized reach as the proportion of quantitative survey respondents reporting participation in two primary RISE participatory design activities." This would not be satisfactory if the survey procedure shares the same limitations in reaching the population (such as those who have to work, or those with different language preferences). It will be helful to report the strategies of recruitment for the survey, how it is different from invitation to participate, and the participation rate of the survey (as a proportion of the total number of intended/targeted population).
4. The authors reported (p. 15) that "For Suva participatory design workshops, respondents living with disabilities were 92% less likely to report participating compared to respondents without disabilities." It would mean that there is a super high rate of disability in Suva; please check for accuracy.
5. In assessing the community member's level of satisfaction (dose), is it possible to separate into steps, such as information received before the meeting, clarity about the agenda/purpose of the meeting, clarity of information during the meeting, and the arrangement of participation (the rules of the proceeding was clear and encouraging participation), etc.?
6. Please give examples of how the participants may perceive that they have had "influenced on decision-making" (i.e., which decisions, who made the decision, how was the decision made [e.g., was there voting], was the

influence observable during the consultation). It is especially difficult to understand those who “would have preferred less influence”, without given the context. For example, they may have chosen not to attend, or not to speak during the meeting.
