# Supplementary material for: Who participates in ‘participatory design’ of WASH infrastructure: A mixed-methods process evaluation
Source: PLOS Glob Public Health. 2025 Jun 13;5(6):e0003430. doi: 10.1371/journal.pgph.0003430 (PMC12165399; doi:10.1371/journal.pgph.0003430)
Supplement: Attachment — Submitted filename: Responses to Reviewers_2025-01-29.docx [file pgph.0003430.s008.docx]

**RE: Who participates in ‘participatory design’ of WASH infrastructure: a mixed-methods process evaluation (PGPH-D-24-01162)**

To Whom it May Concern,

We are grateful to the editor and the reviewers for the time spent reviewing this manuscript.

Please find our responses to all comments below, which were helpful in improving the quality of this manuscript. We have uploaded both ‘tracked changes’ and ‘clean’ versions of the manuscript to facilitate easier re-review. We hope these revisions are acceptable, and please contact me with any questions.

Thank you for your consideration, and we look forward to hearing from you.

On behalf of all authors,

Thea Mink

**Responses to Review Comments**

# **Manuscript: Who participates in ‘participatory design’ of WASH infrastructure: a mixed-methods process evaluation (PGPH-D-24-01162)**

| **Journal Requirements** | | |
| --- | --- | --- |
| Comments/requested revisions | Authors’ response | Additions/edits to manuscript (as applicable), with new text underlined |
| **Additional Editor Comments (if provided):** | | |
| E.1  Please provide a summary (with timelines, if possible) of the different Community engagement activities conducted as part of the RISE trial. This can be included as a figure & will help contextualize the findings presented in this manuscript. | E.1  Thank you for this recommendation. We included a brief summary of the two primary RISE participatory design activities (participatory design workshops and household visits). Additionally, we included a timeline (Fig 1) that includes the dates of primary participatory design activities and data collection activities (surveys, in-depth interviews, and focus group discussions). | E.1  The following text was added:  “Participatory design workshops included mapping of important community features, discussions of upgrading design options, and markings of potential infrastructure [added citation ADB and RISE, 2019]. Household visits discussed individual household conditions and preferences for infrastructure connection [added citation ADB and RISE, 2021]. Further information about the RISE program and participatory design process is outlined elsewhere [[18,26, added citation Francis et al., 2023]](https://www.zotero.org/google-docs/?gV283k).  Fig 1 has been uploaded as a separate file in this re-submission. Fig 1 caption was added to manuscript text:  “Fig 1. Timeline of primary RISE participatory design activities (participatory design workshops and household visits) and data collection activities.”  Text was revised to include the end dates of both participatory design workshops and household visits:  “Participatory design activities, conducted by RISE staff, commenced soon after the baseline surveys in each country and continued through October 2019 in Makassar and December 2020 in Suva **(Fig 1)**.” |
| E.2  The findings of the multivariable logistic regression analysis (Tables 2, 4 & 5) can be combined into a single table. If the analysis was conducted separately for Makassar and Suva, please present the results separately, either within the main text or as supplementary material. | E.2  We have combined Tables 4 and 5 into one table as they both relate to Suva findings; however, we have retained Table 2 as a separate table as the data relate to a separate research question.  All analyses were conducted separately for Makassar and Suva. This is noted in our methods section:  “Analysis was conducted separately by country because of differences in intervention timelines and socio-cultural contexts.”  To further reflect this analysis, both the Table 2 results and related main text for Makassar and Suva are presented separately, with Makassar results for participation reported first and Suva results for participation reported second. | E.2  The following title text was added to the revised Table 4:  “Table 4. Parameter estimates from multivariable logistic regression models of socio-demographic predictors of influence discordance among Suva participatory design participants.” |
| **Reviewer #1: Reviewer Comments** | | |
| Summary |  |  |
| R1.1  The authors aimed to assess reach and satisfaction of WASH interventions in selected communities. The article is well written and spells out clearly the research questions and research process. The methodology is rigorous and justifiable. The paper also makes informed recommendations for future programming. | R1.1  We appreciate the reviewer’s time and attention to providing feedback for this manuscript. We have responded to each comment and have added edits to the manuscript as applicable. | R1.1  N/A |
| Major Issues |  |  |
| R1.2 No major issues of concern were identified. |  |  |
| Minor Issues |  |  |
| R1.3  The authors, in the bid to assess dose received asked all study participants “how much influence respondents and their households felt 142 they had over RISE decision-making, along with how much influence respondents and their 143 households would have preferred”. These questions were asked regardless of whether subjects participated in the RISE intervention or not. This begs the question why study participants who did not contribute to the intervention were still included. Was this to find out their preferred influence had they participated. If this is so, then must be clarified. Furthermore, how will you be able to classify this group then as matched or discordant, since the ‘experienced’ component is unavailable to do the comparison the authors describe. | R1.3  Reviewer 1 is correct in that questions regarding "influence" were asked of all survey participants; however, for our analysis of dose received, the analytic sample was restricted to those who said that they participated in one or both primary RISE participatory design activities. Therefore, those who did not participate in any RISE participatory design activities were not included in analysis. Dose received was first operationalized on page 7, and we have added further clarification in the methods section on page 9. | R1.3  The following text was added:  “Satisfaction with RISE decision-making was determined by comparing participants’ experienced level of influence (none, a little, a lot) to participants’ preferred level of influence (none, a little, a lot). Satisfaction was calculated only for participants who reported that they had participated in at least one of the primary RISE participatory design activities.  In Makassar, when comparing preferred and experienced levels of influence among participants of at least one primary RISE participatory design activity, 161 (66%) of 245 were satisfied with their level of influence, 51 (21%) would have preferred more influence, and 33 (13%) would have preferred less influence (**S4 Table**). Among Suva participants of at least one primary RISE participatory design activity, 207 (70%) of 297 were satisfied with their level of influence, 27 (9%) would have preferred more influence, and 63 (21%) would have preferred less influence (**S5 Table**). |
| R1.4  The authors could also clarify why the p-value significant level was unconventionally set at 0.25. | R1.4  The p-value of 0.25 was used during bivariate analysis to build the full models. However, we used a conventional p-value of 0.05 as a threshold for ‘significant’ findings from the full models.  The p-value of 0.25 for model building follows recommendations from Bursac et al.'s for purposeful selection of logistic regression covariates, as a p-value of 0.05 can fail to identify variables that are known to be important (2008). This citation was included in our text, “Predictor variables were selected for inclusion in full models if their coefficient p-value was less than 0.25 (Bursac et al., 2008).” | R1.4  N/A |
| **Reviewer #2 Comments** | | |
| R2.1  The manuscript described a mixed-method approach to evaluate the participation by community members of RISE programs in two countries, with the intention to identify factors and barriers affecting participation. The report on this process evaluation is interesting and of importance, but several clarifications could be made to facilitate reader’s understanding of the context. | R2.1  We appreciate the reviewer’s time and effort to provide feedback for this manuscript. We have responded to each comment and have added edits to the manuscript as applicable. | R2.1  N/A |
| R2.2  To provide a background and context to the study, please give a description of the following concerns so that readers may get a general idea about the motivation to participate. | R2.2  Thank you for the sub-comments regarding background context (R2.2.i - R2.2.iv.). We have responded below. | R2.2  N/A |
| R2.2.i  What is the relative importance of this project among similar development programs in recent years? | R2.2.i  We included context on other interventions in the text. Further information on the relative importance of the RISE trial can be found in the RISE trial protocol paper cited in the text (Leder et. al., 2021). | R2.2.i  We added the following text:  “The RISE intervention includes nature-based and decentralized components (pressure tanks, constructed wetlands, and rainwater tanks) to decrease exposure to fecal contamination. These components were selected because conventional water and sewer systems do not often service informal settlements, and the evidence is mixed for other lower-cost water and sanitation interventions [18, added citations 19-25].” |
| R2.2.ii  What is the relationship between the agents of implementation and agents conducting the participatory processes? | R2.2.ii  The participatory design process was a component of the overall RISE trial. Therefore, during the design phase, the ‘agents of implementation’ and the ‘agents conducting the participatory processes’ were the same. The implementation of the participatory design processes were conducted by RISE staff. We added clarifying language in the text. | R2.2.ii  We added the following text on page 6:  “Participatory design activities, conducted by RISE staff, commenced soon after the baseline surveys in each country and continued through October 2019 in Makassar and December 2020 in Suva **(Fig 1)**.” |
| R2.2.iii  What is the perception of local residents regarding the two questions above? What would motivate people to participate? | R2.2.iii  The perception of local residents regarding the two questions above and residents’ motivation to participate are addressed in another paper. Specifically, they are addressed in a companion paper by Francis et al. (2023), which is a qualitative implementation science manuscript that assessed key mechanisms for participant inclusion during the RISE participatory design phase. The Francis et al. paper addresses the relative advantage of the RISE program compared to others (R2.2.i), perceptions of the agents of implementation (R2.2.ii), participant motivation to participate in the RISE participatory design phase (R2.2.iii), and perceived need for a water-sanitary service (R2.2.iv).  We have briefly summarized these points in the introduction and have referred readers to more detail in Francis et al. (2023). | R2.2.iii  We added the following text:  “Specifically, participatory design workshops and household visits were planned to reach a diverse population (with a particular focus on marginalized groups and women) and to meaningfully collaborate with participants in intervention decision-making [[26,27]](https://www.zotero.org/google-docs/?X6KrIG). The participatory design activities helped identify and adapt to residents’ needs, which included solutions to environmental stressors, and residents were motivated to participate because of RISE’s emphasis on inclusion [added citation Francis et al., 2023]. Participatory design workshops included mapping of important community features, discussions of upgrading design options, and marking out potential infrastructure locations [added citation ADB and RISE, 2021]. Household visits discussed individual household conditions and preferences for infrastructure connection [added citation ADB and RISE, 2021]. Further information about the RISE program and participatory design process is outlined elsewhere [[18,26, added citation Francis et al., 2023]](https://www.zotero.org/google-docs/?gV283k).  “Along with the movement towards comprehensive WASH interventions, there are calls for more comprehensive evaluation of such interventions [[12,13,28,29]](https://www.zotero.org/google-docs/?broken=WHu6dC). Towards that goal, we conducted two companion studies relating to the RISE participatory design phase: one on implementation science mechanisms for inclusion and the present process evaluation [added citation: Francis et al., 2023].” |
| R2.2.iv  Please report on local residents’ interest in the RISE program and perceived need for a water-sanitary service? | R2.2.iv  Please see response in R2.2.iii. | R2.2.iv  Please see additions to manuscript in R2.2.iii. |
| R2.3  Related to the previous comment, the authors did not explain how the RISE program is going to have impact on the use of land, and how this was communicated with the residents before? | R2.3  We believe that further elaboration on land use is outside the scope of our paper, however, we have cited additional resources that outline RISE’s impact on land use and communication with residents. | R2.3  We added the following citation:  “Further information about the RISE program and participatory design process is outlined elsewhere [[18,19, added citation Francis et al., 2023](https://www.zotero.org/google-docs/?broken=z9euV6)].” |
| R2.4  In assessing reach (p. 6): “We operationalized reach as the proportion of quantitative survey respondents reporting participation in two primary RISE participatory design activities.” This would not be satisfactory if the survey procedure shares the same limitations in reaching the population (such as those who have to work, or those with different language preferences). It will be helpful to report the strategies of recruitment for the survey, how it is different from invitation to participate, and the participation rate of the survey (as a proportion of the total number of intended/targeted population). | R2.4  We added recruitment strategy background for the overall RISE trial, and cited Leder et al., 2021, the trial protocol paper, for further information.  The survey recruitment was described in the methods text as follows: “This process evaluation used quantitative data from surveys that targeted all households that had previously enrolled and consented to RISE. Two adult survey respondents, one man and one woman, were targeted from each household.” We added further clarification about recruitment non-response in the methods section.  In addition, we added survey participation context in the methods section and we reported the full survey participation rate (for both intervention and control settlements) to the results section. | R2.4  We added the following text to the methods:  “RISE attempted to enroll all households in each settlement, and enrollment and baseline surveys took place in late 2018 in Makassar and mid-2019 in Suva [[18]](https://www.zotero.org/google-docs/?avtnfx).”  “This process evaluation used quantitative data from surveys that targeted all intervention and control households that had previously enrolled and consented to RISE in Makassar (11 settlements, 593 households) and Suva (12 settlements, 767 households).”  “All teams collected data on tablets equipped with the SurveyCTO application. Per RISE protocols, individuals were considered a non-response for the survey if they were not reached after three phone calls in Makassar and after two house visits in Suva. Further information on the methods for the quantitative survey design and administration can be found elsewhere [[35]](https://www.zotero.org/google-docs/?broken=1L8SmH).”  We added the following text to the results:  “The response rate for the survey was 66% in Makassar (1,172 targeted) and 92% in Suva (1,308 targeted). Our analytic sample, which included only those in intervention settlements, consisted of 320 surveys (185 with women and 135 with men) from five sites in Makassar and 503 surveys (254 with women and 249 with men) from six sites in Suva.” |
| R2.5  The authors reported (p. 15) that “For Suva participatory design workshops, respondents living with disabilities were 92% less likely to report participating compared to respondents without disabilities.” It would mean that there is a super high rate of disability in Suva; please check for accuracy. | R2.5  The sample size for those reporting living with disability was small in Suva (n=9). Given this, we cautioned interpretation of this finding in our discussion:  “In Suva, household visits appeared to be more inclusive of people living with disabilities than community-level workshops, although this interpretation may be limited by sample size.”  We have updated the odds ratio interpretation in the text for further clarity. | R2.5  We changed the following text:  “For Suva participatory design workshops, the odds of participation were 92% lower for respondents living with disabilities compared to respondents living without disabilities (95% CI: 0.0, 0.8) (**Table 2**).  To better align with the above revised text, we also updated the following text in the results:  For Makassar participatory design workshops, the odds of participation were 2.6 times higher for women compared to men (95% confidence interval (CI): 1.8, 3.8) and 2.1 times higher for married respondents compared to respondents with other marital statuses (95% CI: 1.3, 3.4).  For Makassar household visits, the odds of participation were 3.6 times higher for women compared to men (95% CI: 1.4, 9.5).  For Suva household visits, the odds of participation were 1.7 times higher for people who completed secondary education or above compared to those who completed primary education or below (95% CI: 1.2, 2.5).  For Suva participatory design participants, the odds of wanting *more* influence than what was experienced were 3.3 times higher for women compared to men (95% CI: 1.2, 8.8) (**Table 4**).  The odds of wanting *less* influence than what was experienced were 2.6 times higher for religious minority respondents compared to Christian religious majority respondents (95% CI: 1.3, 5.1) and were 1.8 times higher for unmarried respondents (including single, divorced, or widowed) compared to married respondents (95% CI: 1.2, 2.9) (**Table 4**). |
| R2.6  In assessing the community member’s level of satisfaction (dose), is it possible to separate into steps, such as information received before the meeting, clarity about the agenda/purpose of the meeting, clarity of information during the meeting, and the arrangement of participation (the rules of the proceeding was clear and encouraging participation), etc.? | R2.6  Unfortunately, we are not able to separate the components of satisfaction (dose) with influence over RISE decision-making in the quantitative data. Our secondary analysis leveraged two survey questions that broadly asked about influence. Participants interpreted these questions as they saw fit for their own experiences and preferences.  The below questions are included in the S1 Table:  Question 1 about experienced influence asked, “How much influence do you feel you and your household had over the RISE-related decisions that will affect your household, cluster, or settlement?”  Question 2 about preferred influence asked, “How much influence would you and your household have preferred to have over the RISE-related decisions that will affect your household, cluster, or settlement?” | R2.6  N/A |
| R2.7  Please give examples of how the participants may perceive that they have had “influenced on decision-making” (i.e., which decisions, who made the decision, how was the decision made [e.g., was there voting], was the influence observable during the consultation). It is especially difficult to understand those who “would have preferred less influence”, without given the context. For example, they may have chosen not to attend, or not to speak during the meeting. | R2.7  We have included examples of decision-making opportunities during the participatory design phase in the introduction section. | R2.7  We added the following text:  “Specifically, participatory design workshops and household visits were planned to reach a diverse population (with a particular focus on marginalized groups and women) and to meaningfully collaborate with participants in intervention decision-making [[26,27]](https://www.zotero.org/google-docs/?X6KrIG). The participatory design activities helped identify and adapt to residents’ needs, which included solutions to environmental stressors, and residents were motivated to participate because of RISE’s emphasis on inclusion [added citation Francis et al., 2023]. Participatory design workshops included mapping of important community features, discussions of upgrading design options, and markings of potential infrastructure [added citation Prescott et al., 2021]. Household visits discussed individual household conditions and preferences for infrastructure connection [added citation ADB and RISE, 2021]. Further information about the RISE program and participatory design process is outlined elsewhere [[18,26, added citation Francis et al., 2023]](https://www.zotero.org/google-docs/?gV283k). |
|  |  | In addition to the above changes to the manuscript text in response to reviewer comments, we updated all quotes to identify whether they were from participants in Makassar or Suva for further clarity. For example:  *I usually came to represent my family because my husband was working.* (IDI, Woman, Makassar) |
