# Supplementary material for: Who participates in ‘participatory design’ of WASH infrastructure: A mixed-methods process evaluation
Source: PLOS Glob Public Health. 2025 Jun 13;5(6):e0003430. doi: 10.1371/journal.pgph.0003430 (PMC12165399; doi:10.1371/journal.pgph.0003430)
Supplement: Attachment — Submitted filename: Response to Reviewers_2025-05-09.docx [file pgph.0003430.s009.docx]

**RE: Who participates in ‘participatory design’ of WASH infrastructure: a mixed-methods process evaluation (PGPH-D-24-01162)**

To Whom it May Concern,

We are grateful to the editor and to the reviewers for their time reviewing the manuscript. Please find our responses to all comments below.

We have uploaded both ‘tracked changes’ and ‘clean’ versions of the manuscript to assist re-review. We hope these revisions are acceptable, and please contact me with any questions.

Thank you for your consideration, and we look forward to hearing from you.

On behalf of all authors,

Thea Mink

| **Journal Requirements** | | |
| --- | --- | --- |
| Comments/requested revisions | Authors’ response | Additions/edits to manuscript (as applicable), with new text underlined |
| **Additional Editor Comments** |  |  |
| In addition to the minor comments from Reviewer 1, please also address the following comments: |  |  |
| E.1  Were the data collectors for the quantitative surveys and the qualitative interviews involved with trial implementation? If yes, could their involvement have introduced reporting bias? | E.1  Yes, it is possible that the data collectors for the quantitative surveys and qualitative interviews could have introduced some reporting bias. While the data collection team was not involved with trial implementation, they were affiliated with the larger RISE trial as members of the assessment team.  We have noted this in the limitations section. | E.1  Data collectors for the quantitative and qualitative data may have introduced reporting bias; while they were not directly involved in RISE implementation, they were affiliated with the larger RISE trial. |
| E.2  Tables 1 and 3: Please present the percentages (%) in parenthesis. Also, for Table 1, kindly highlight that the age was in completed years. | E.2  We have updated the Table 1 and 3 percentage formatting and added age in years. | E.2  Age (years)  Please see Tables 1 and 3 for changes. |
| E.3  Tables 2 and 4: For ease of reading, please separate the lower & upper bound confidence intervals with a coma (,). | E.3  Changes made in manuscript. | E.3  Please see Tables 2 and 4 for changes. |
| E.4  Page 16: “For every one year increase in age in years, the adjusted odds of participation increased by 3% (95% CI: 1.0, 1.0).” The upper-bound confidence interval, as presented gives a false impression that it is same as the lower-bound confidence interval (possibly due to rounding) – kindly review & update, as needed. | E.4  We included the confidence interval’s hundredths decimal place for clarity. | E.4  For every one year increase in age in years, the adjusted odds of participation increased by 3% (95% CI: 1.02, 1.03). |
| **Reviewer #1: Reviewer Comments** |  |  |
| R1.1  31 Is the one billion people exact? You may say approximately; also as you are referring to people use ‘who’ instead of ‘which’. This is the opening sentence of your work so should not attract any ambiguity. | R1.1  Thank you for your comment. Changes made in manuscript. Additionally, we updated the citation for this statistic to reflect the current 2024 SDG report. | R1.1  1.1 billion people live in urban informal settlements and regularly contend with unimproved water and sanitation services (including poor access, quality, and reliability), in addition to insufficient living areas, poor housing durability, and unstable land tenure [1-3; updated citation 3 to UN DESA]. |
| R1.2  83 Consider ‘few’ in place of ‘handful’ | R1.2  Changes made in manuscript. | R1.2  Among the several water and sanitation process evaluations in the literature, only a few have identified factors that affected participation and assessed participant satisfaction with interventions [[6,32–34]](https://www.zotero.org/google-docs/?cTT4MR). |
| R1.3  Clear methodology and good analyses from results Overall, an impressive addition to knowledge | R1.3  Thank you for your thorough review and feedback. | N/A |
| **Reviewer #2: Reviewer Comments** |  |  |
| R2.1  The authors have responded to my comments, some of them by referring to a previous manuscript published by the research team. | R2.1  Thank you for your thoughtful feedback throughout the review process. | N/A |
| **Amended citations**  We reviewed all citations for retractions and found none. The following change for citation accuracy. |  |  |
| C.1 | C.1  We updated the citation [2] [Schrecongost A, Wong](https://www.zotero.org/google-docs/?GFADAj) link to PDF, as the original link did not connect. | C.1  [Schrecongost A, Wong K. Unsettled: Water and sanitation in urban settlement communities in the Pacific [Internet]. World Bank; 2015. Available from: http://documents.worldbank.org/curated/en/603081468197054598](https://www.zotero.org/google-docs/?GFADAj) |
